# Alterations of Liver Morphology in Senescent Rats

**DOI:** 10.3390/ijms25189846

**Published:** 2024-09-12

**Authors:** Ruth Maldonado-Rengel, Zaida Sócola-Barsallo, Bélgica Vásquez

**Affiliations:** 1 Doctoral Program in Morphological Sciences, Faculty of Medicine, Universidad de La Frontera, Avenida Francisco Salazar 01145, Temuco 4811230, Chile; remaldonado6@utpl.edu.ec; 2 Department of Health Sciences, Universidad Técnica Particular de Loja, San Cayetano Alto, Calle París, Loja 110107, Ecuador; zesocola@utpl.edu.ec; 3 Department of Basic Sciences, Faculty of Medicine, Universidad de La Frontera, Avenida Francisco Salazar 01145, Temuco 4811230, Chile; 4 Centre of Excellence in Morphological and Surgical Studies, Universidad de La Frontera, Avenida Francisco Salazar 01145, Temuco 4811230, Chile

**Keywords:** liver, senescence, morphological changes

## Abstract

Age-related liver changes can have important implications for health and metabolic function. This study aimed to describe the morphoquantitative alterations of the liver in senescent rats compared to adult rats. Twelve male rats were used, divided into 6-month-old adults (group A) and 36-month-old senescent rats (group S). Morphometric and histopathological studies, quantification of collagen types I and III, and stereological analyses were performed to determine the volume density of mononucleated (Vv_hepM_) and binucleated (Vv_hepB_) hepatocyte nuclei, surface area density (Sv_hepM_), and number density (Nv_hepM_) of mononucleated hepatocyte nuclei. The findings reveal an alteration of the normal liver tissue architecture in senescent rats and the presence of inflammatory lesions and fibrosis. In addition, there was a decrease in body and liver mass and volume. Group S showed a significant reduction in Vv_hepM_ and Nv_hepM_; however, Sv_hepM_ was significantly higher. No significant differences were noted in the percentage of binucleated hepatocytes between the two groups. This study reveals substantial morphological changes in the aging liver, with possible functional implications. More research is needed on the underlying mechanisms and their consequences at older ages.

## 1. Introduction

The liver is a vital organ in mammalian physiology, playing crucial roles in metabolism, detoxification, plasma protein synthesis, and nutrient storage [1]. Its correct functioning is essential to maintaining organismal homeostasis. Rats (Rattus norvegicus) are often used as experimental subjects in scientific research to examine physiological and pathological mechanisms owing to their biological and genetic similarities to humans [2].

Aging or senescence is a natural process that leads to a progressive decline in physiological function and increased vulnerability to disease. Using senescent rats as models to study aging in mammals provides valuable information on age-related changes occurring in various organs, including the liver. Studying liver alterations in senescent rats is of great importance for understanding age-related liver diseases in humans, such as fibrosis, cirrhosis, and liver cancer [3,4,5].

Numerous studies have characterized liver morphology in young and adult rats, establishing a reference framework for identifying aging-related changes. The liver presents a well-organized architecture in young rats with uniformly sized hepatocytes and a regular sinusoidal network [6]. The liver undergoes noticeable morphological changes as it ages, such as the buildup of lipofuscin, which indicates increased oxidative stress and fibrosis. These changes are linked to a decline in the regenerative capacity of the liver [7,8,9]. Other studies have reported alterations in the structure of the endoplasmic reticulum and mitochondria, suggesting mitochondrial dysfunction and reduced efficiency of protein processing [10]. Liver sinusoidal endothelial cells also thicken, and their fenestrations decrease. This, along with impaired endocytosis and increased leukocyte adhesion, reduces hepatic perfusion and affects lipoprotein metabolism [11,12].

A recent study by Baiocchi et al. [5] highlights how aging affects liver cells and contributes to the development of liver diseases, focusing on the biliary and vascular compartments. The results show that aging leads to dysfunctions in cell proliferation, increased oxidative stress, and changes in the hepatic microvasculature, which combined promote the progression of fibrosis and cirrhosis and, eventually, liver cancer.

Several biological mechanisms are believed to contribute to age-related hepatic alterations. Mitochondrial dysfunction is one of the main factors leading to increased oxidative stress and accumulation of cellular damage [13]. In addition, activation of chronic inflammatory responses may contribute to liver tissue degeneration and the development of fibrosis [9]. Cell signaling and DNA repair capacity changes also play a crucial role in hepatic aging [14]. The aging process involves a decline in the liver’s ability to metabolize drugs and toxins, increasing susceptibility to liver diseases [4].

Despite advances in understanding age-related hepatic morphological changes, knowledge gaps remain, especially in the detailed characterization of these alterations in senescent rats. Considering the above, this study aimed to describe the morphoquantitative alterations of the liver in senescent rats compared to adult rats. A better understanding of these alterations has important clinical and biomedical implications. It could contribute to developing strategies to prevent and treat age-related liver diseases, thereby improving the quality of life of the aging population [8].

## 2. Results

### 2.1. Descriptive Histopathological Analysis of Rat Liver Reveals Key Differences between Adult and Senescent Specimens

**Figure 1:**
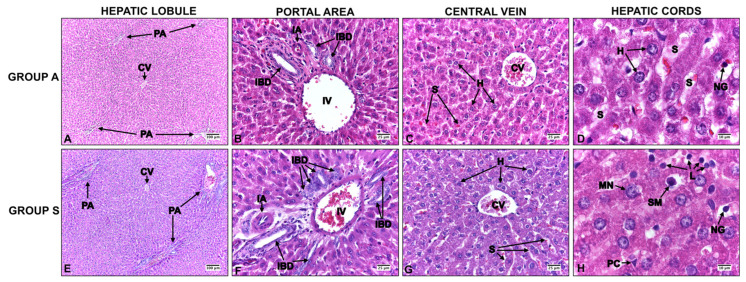
Liver of adult Sprague Dawley rats (group A) (**A**–**D**) versus senescent rats (group S) (**E**–**H**). PA: portal area; CV: central vein, IA: interlobular artery; IV: interlobular vein; IBD: interlobular bile duct; H: hepatocyte; S: sinusoid; SM: stellate macrophage; MN: macronucleus; PC: perisinusoidal cell; L: leukocytes; NG: neutrophilic granule. H&E staining.

In group A, the liver was histologically characterized by a lobular structure that did not present the classic hexagonal shape. Several large veins were observed in its structure, some of which were central and other interlobular veins in the portal spaces. The portal space, surrounded by a continuous row of hepatocytes, contained stromal lax connective tissue and the hepatic triad, composed of the artery, vein, and interlobular bile duct lined by cuboidal cells. Between one and two biliary ducts were seen in each portal space, in addition to peribiliary lymphatic vessels. Occasionally, segmental lymphocytes and leukocytes were visualized in the connective tissue. There were anastomosed cords of hepatocytes between the portal space and the central vein, separated by interconnected sinusoids leading to the central vein. Polyhedral-shaped hepatocytes were observed on higher magnification. The large, pale, centrally located nuclei were clearly defined by a thin border of violet-blue-stained chromatin near the nuclear membrane. Large, dark nucleoli were evident. There was also variation in the size of the nuclei, and some hepatocytes were binucleated (Figure 1A–D).

In contrast, a loss of the normal architecture of the liver tissue was observed in group S, with alterations in the cord-like arrangement and formation of hepatocyte clusters in some microscopic fields. The portal spaces frequently showed enlargement, with over three interlobular bile ducts. These ducts were bordered by fibrous tissue, which, in certain instances, extended into the periportal regions. In general, the inflammation was limited to the portal space. The nuclei of the hepatocytes were enlarged, and hyperchromatic and necrotic cells with eosinophilic cytoplasm were identified (Figure 1E–H).

### 2.2. The Liver Tissue of Senescent Rats Shows Inflammatory Lesions and Fibrosis Predominantly Confined to the Portal Space

In group S, the modified Scheuer scale for portal inflammation and interphase hepatitis revealed that 66.7% of the cases presented grade 1, while 33.3% showed grade 2. As for lobular activity, 33.3% showed grade 1 and 66.7% showed grade 2. Regarding the extent of liver fibrosis, 50% presented stage 1 and 16.7% stage 2, while the remaining samples showed no fibrosis. On the other hand, no histopathological alterations were observed in group A.

**Figure 2:**
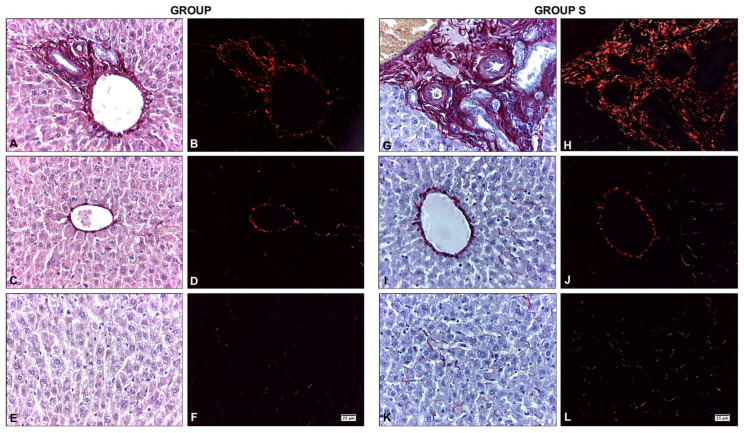
Presence of type I collagen fibers (red) in the liver tissue of adult Sprague Dawley rats (group A) versus senescent rats (group S). (**A**,**C**,**E**,**G**,**I**,**K**), staining with Sirius Red without polarization. (**B**,**D**,**F**,**H**,**J**,**L**), stained with Sirius Red with polarization.

Using Sirius Red staining, the liver samples from both groups showed the presence of types I and III collagen fibers, with a greater prominence in the portal spaces (Figure 2). Assessment of fibrosis by quantitative measurement of collagen revealed significant differences between the groups. Type I collagen content was higher in group S than in group A (*p* = 0.001); however, there was no significant difference between the two groups in type III collagen content (*p* = 0.541; Table 1).

Regarding the morphological changes of the interlobular bile ducts, the Scheuer stage scale of primary biliary cholangitis indicated that 83.3% of the cases in group S were classified as stage 1. In contrast, no alterations in the interlobular bile ducts were noted in group A.

### 2.3. In Senescent Rats, the Reduction of Body and Liver Morphometric Parameters Is Accompanied by Significant Cellular Changes: The Hepatic Nuclei Increase in Size While Their Number Decreases

Significant differences in morphometric parameters were observed between the A and S groups. Senescent rats showed a notable decrease in body mass, liver mass, and volume compared to adult rats (Table 2).

Group S presented a reduction in the volume and number density of mononuclear hepatocyte nuclei, with significantly lower mean values than group A. However, the surface area density was significantly higher. No significant differences were observed in the percentage of binucleated hepatocytes between the two groups (Table 3).

## 3. Discussion

This study describes the morphological and quantitative changes in the liver of senescent rats compared to adult rats. The findings reveal a clear loss of normal liver tissue architecture, inflammatory lesions, fibrosis, reduced body and liver morphometric parameters, and changes in the size and number of hepatocyte nuclei in aging rats.

Our data revealed the presence of portal and lobular inflammation in senescent rats, which is consistent with the activation of chronic inflammatory responses reported in other studies [9]. Chronic inflammation in the aging liver can be attributed to several biological factors. One of the main mechanisms is mitochondrial dysfunction, leading to increased oxidative stress and accumulation of cellular damage [10,13]. The imbalance between reactive oxygen species (ROS) and reactive nitrogen species (RNS) plays a crucial role in this process, as the excessive production of ROS/RNS leads to oxidative stress, which further damages cellular components, contributing to liver inflammation and fibrosis [15]. Mitochondrial dynamics, including fission and fusion processes, are also altered in aging livers, exacerbating oxidative stress and disrupting cellular homeostasis [16].

Another important mechanism is the activation of the senescence-associated secretory phenotype (SASP). Senescent cells secrete a variety of proinflammatory cytokines, growth factors, and proteases that contribute to an inflammatory microenvironment [17,18,19,20]. The SASP is characterized by the secretion of multiple signaling molecules that include interleukins (e.g., IL-6, IL-8), chemokines, and matrix metalloproteinases (MMPs). These factors not only perpetuate cellular damage by maintaining a state of chronic inflammation but also actively remodel the tissue environment. This remodeling can promote fibrosis by stimulating the proliferation and activation of fibroblasts and hepatic stellate cells, leading to excessive deposition of extracellular matrix components such as collagen [17,19].

Moreover, the SASP factors are known to reinforce the senescent state both in an autocrine and paracrine manner, creating a self-sustaining loop that exacerbates tissue dysfunction and senescence spread to neighboring cells [18,20]. The inflammatory milieu created by SASP also contributes to the disruption of normal tissue architecture and function, further accelerating the decline in liver function observed in aging [20]. Importantly, the nature and intensity of the SASP can vary depending on the cell type and the stressor that induced senescence, highlighting the complex role of SASP in both promoting tissue repair and contributing to pathological conditions such as fibrosis and liver dysfunction [17]. Ogrodnik et al. [21] highlighted that SASP secretion could lead to cell cycle retention and increased ribosome biogenesis, resulting in lower-quality protein production and increased oxidative stress.

On the other hand, portal inflammation, in particular, may be linked to the liver’s adaptive response to microvascular dysfunction. This dysfunction is frequently driven by damage to the endothelial cells lining the blood vessels, which plays a critical role in regulating vascular tone. Endothelial damage disrupts the production of nitric oxide (NO), a key molecule responsible for inducing vasodilation, the process by which blood vessels widen to increase blood flow. When NO production is imbalanced, the blood vessels are unable to dilate properly, leading to sustained vasoconstriction. This impaired vasodilation not only restricts blood flow but also exacerbates pressure within the liver’s microvasculature, contributing significantly to hepatic microvascular dysfunction. As a result, the liver’s ability to efficiently perfuse and oxygenate its tissues is compromised, potentially leading to further liver damage and inflammation [22,23]. Maeso-Díaz et al. [24] reinforced this idea by showing that impaired microcirculatory function and a change in sinusoidal phenotype can influence adaptive responses in the aging liver, exacerbating fibrosis and inflammation. For their part, Baiocchi et al. [5] showed that aging affects the hepatic microvasculature, which may reduce hepatic perfusion and contribute to the inflammation and fibrosis seen in senescent rats.

Regarding liver fibrosis, our findings align with previous studies [8,9]. They reveal a significant buildup of fibrosis in senescent rats, with a predominant distribution in portal spaces and, to a lesser degree, in periportal regions. This fibrosis, classified in stages 1 and 2 according to the Scheuer scale, indicates a critical imbalance between the production and degradation of the extracellular matrix, a phenomenon that triggers increased fibrinogenesis [9]. The liver fibrosis observed is characterized by excessive synthesis and reduced degradation of extracellular matrix components, mainly collagen. This process alters the liver’s normal architecture, increasing tissue stiffness and promoting the activation of hepatic stellate cells, considered the main mediators of fibrogenesis [25]. The imbalance in extracellular matrix homeostasis perpetuates fibrosis and can lead to progressive liver dysfunction [9]. The buildup of fibrotic tissue compromises the hepatic lobular structure, potentially affecting portal blood flow and hepatocyte function, which could eventually result in decreased liver function [26]. Importantly, the presence of fibrosis at early stages (1 and 2) in senescent rats suggests that aging per se may contribute to the development of liver fibrosis, possibly due to the accumulation of oxidative damage, cellular senescence, and chronic inflammation associated with age.

Furthermore, there was a rise in the number of interlobular bile ducts and observed alterations in their structure, which align with the early phase of primary biliary cholangitis as per the Scheuer scale [27]. These findings are consistent with the study by Baiocchi et al. [5], who note how aging affects the biliary compartment and contributes to the development of chronic liver diseases.

A significant finding of our study was the increased size of hepatocyte nuclei in senescent rats, evidenced by a higher surface density of mononuclear hepatocyte nuclei (Sv_hepM_), which was significantly higher (*p* = 0.001) in senescent rats than in adult rats. These results are consistent with previous studies that have reported an increase in hepatocyte nuclear size during aging, both in human [28,29] and animal models [30]. This finding suggests an increase in relative cell surface area, which could indicate increased compensatory cellular activity in response to senescence [14]. The increase in nuclear size could be related to several age-related factors, including gene expression changes, chromatin alterations, and a reduced DNA repair capacity [14,30]. Neurohr et al. [18] reported that cell overgrowth is characterized by cytoplasm dilution, which leads to senescence. This occurs because the cell cannot adjust nucleic acid and protein biosynthesis to match the increasing cell volume. In this vein, Ge et al. [31] describe hepatic senescent cells as exhibiting distinctive morphological features, such as an increase in cell size, the presence of cytoplasmic vacuoles, and changes in the structure of the nucleus, including enlargement and changes in heterochromatin.

Additionally, the observed morphometric changes and lower volume density (Vv_hepM_) and number (Nv_hepM_) of mononuclear hepatocytes can be explained by several mechanisms associated with cellular aging and senescence. In aging, cells experience telomere shortening and DNA damage, leading to cell cycle arrest and a decrease in the proliferative capacity of the hepatocytes [32,33]. In addition, apoptosis increases, further reducing cell numbers [34]. This decreased proliferation and increased apoptosis result in a lower volume density and number of hepatocytes. These findings underscore the importance of cellular senescence in the morphological and functional alteration of the liver during aging, providing evidence of significant changes in hepatocyte structure.

Despite the significant findings, this study has some limitations. First, the sample was limited to a single animal model, so extrapolating these results to humans should be conducted with caution. Second, the analysis focused mainly on morphological and structural changes without comprehensively assessing functional changes. Furthermore, evaluating the molecular mechanisms underlying the observed alterations could provide a deeper understanding of hepatic aging processes.

Future studies should prioritize the investigation of the intricate molecular pathways that contribute to chronic inflammation in the aging liver. Studies analyzing the interaction between mitochondrial dysfunction, oxidative stress, and the inflammatory response could provide a more comprehensive understanding of these processes. In addition, exploring therapeutic interventions that can mitigate SASP and reduce inflammation could present novel opportunities to manage age-related liver diseases.

## 4. Materials and Methods

### 4.1. Animals

Experimental procedures were performed according to the Guide for the Care and Use of Laboratory Animals [35], and the experimental protocol was approved by the Scientific Ethics Committee of the Universidad de La Frontera, Chile (File Number 041_24). Twelve male Sprague Dawley rats were divided into six 6-month-old adult rats (group A) and six 36-month-old rats (group S). All rats were obtained from the vivarium in the Center of Excellence in Morphological and Surgical Studies (CEMyQ), Universidad de La Frontera, Chile. The rats were kept in controlled environmental conditions regarding temperature, environmental noise, and a cycle of 12 h light/12 h darkness. In addition, they received food and water ad libitum. The choice of male rats is justified because hormonal differences between sexes may influence the physiological and morphological response of the liver. Previous studies have shown that female rats can exhibit hormonal variations that affect the interpretation of results [36]. Furthermore, employing only one gender eliminates an additional variable and enables a more precise evaluation of the impact of age on liver alterations [37].

The sample size determination was based on the bioethical principles of Russell and Burch’s 3 Rs [38] for animal experimentation (replacement, reduction, and refinement) using the minimum number needed to achieve a significant difference [39].

The mass of each animal was recorded before euthanasia, which was performed using an overdose of 160/20 mg/kg ketamine/xylazine [40]. Then, the liver was dissected to determine its mass using an analytical balance (A&D Orion^®^ HR 120, A&D Technology, Saitama, Japan) and volume using the Scherle method [41]. Several liver fragments were immersed in fixative (1.27 mol/L formaldehyde in 0.1 M phosphate buffer pH 7.2) for 48 h at room temperature.

### 4.2. Histological Processing and Staining

Once the samples were fixed, they were dehydrated and embedded in Paraplast Plus (Sigma-Aldrich Co., St. Louis, MO, USA). Once the blocks were obtained, 5 μm thick sections were made on a microtome (Leica^®^ RM 2255, Leica Biosystems, Deer Park, IL, USA). Five sections were made from each block for histopathological and stereological analysis. The sections were stained with H&E to examine tissue structure and with Sirius Red to identify and measure collagen fibers of types I and III. The histological images were obtained using a Leica^®^ DM750 microscope with a Leica^®^ ICC50 HD digital camera and projected onto an LCD View Sonic^®^.

### 4.3. Histopathological Analyses

A descriptive analysis of liver tissue was performed on both groups. The modified Scheuer grade and stage scale [27] was used for the semiquantitative analysis of histological sections. The analysis was performed independently by the authors RME and ZSB. Disagreements were discussed until a consensus was reached. This scale is widely used to assess the severity of liver impairment, especially in chronic liver disease. It provides a detailed framework for quantifying both inflammation (grade) and fibrosis (stage) (Table 4). Briefly, grading refers to the scoring of necroinflammatory injury of hepatitis, including the different types and degrees of hepatocellular damage and the location and extent of the inflammatory process. Staging records the extent of fibrosis and changes in structure. The score ranges from 0 (absence of histopathological signs) to 4 (severity and extent of histopathological signs). Although initially developed for humans, the Scheuer grade and stage scale has been adapted for animal research, including rats [42]. Since senescent rats can show signs of chronic liver damage, this scale makes it possible to assess both acute and chronic lesions present in the liver of these rats. To evaluate the morphological changes of the interlobular bile ducts, the Scheuer scale of the stages of primary biliary cholangitis was used [27]. Scheuer’s stages are described in Table 5.

### 4.4. Quantification of Collagen Fibers

To detect collagenous fibers types I and III in the liver, histological sections were stained with Sirius Red F3BA 0.1% *w*/*v* (Sigma-Aldrich Co., St. Louis, MO, USA) for 1 h in saturated aqueous picric acid solution (Merck, Darmstadt, Germany). Then, they were rinsed in 0.01 N hydrochloric acid (Merck, Darmstadt, Germany) for 2 min, washed in distilled water, stained with Harris hematoxylin (Merck, Darmstadt, Germany) for 2 min, and washed in running water. Finally, the sections were dehydrated in ascending alcohols, rinsed in Xylene (Merck, Darmstadt, Germany), and mounted with Entellan^®^ (Merck, Darmstadt, Germany). Histological images were obtained using a Leica^®^ DM750 microscope with a Leica^®^ ICC50 HD camera and projected on a View Sonic^®^ LCD (ViewSonic, Brea, CA, USA). The total area (μm^2^) of types I and III collagen fibers was measured using the Image Pro Premier 9.1 software (Media Cybernetics, Warrendale, PA, USA).

### 4.5. Liver Stereology

For the stereological analysis of the liver, 5 random microscopic fields were observed for each histological slice, and 240 fields were analyzed in total (120 in each group) [43]. The slides were visualized under a Leica^®^ DM2000 LED stereological microscope and photographed with a Leica^®^ MC170 HD digital camera. The 36-point test system designed by STEPanizer^®^ (https://www.stepanizer.com/) was used, and the following parameters were determined: volume density of mononucleated (Vv_hepM_) and binucleated (Vv_hepB_) hepatocyte nuclei, surface density (Sv_hepM_), and number (Nv_hepM_) of mononucleated hepatocyte nuclei. The volume density was estimated using the following formula: Vv = P_P_/P_T_ (100%), where P_P_ is the number of points touching the structure of interest, and P_T_ is the total number of points in the system (36 points). The surface density was evaluated using the equation Sv = 2 × I/L_T_, where I is the number of intersections touching the structure of interest and L_T_ is the total line length of the 36-point test system. The number density was quantified according to the equation Nv = Q^-^/(A_T_ × t), where Q^-^ is the number of observations of the structures of interest counted in a given area, including the forbidden lines and the forbidden plane, A_T_ is the total area of the test system, and (t) is the thickness of the dissector. The slides were viewed under a Leica^®^ DM2000 LED stereomicroscope and photographed with a Leica^®^ MC170 HD digital camera.

### 4.6. Statistical Analysis

Descriptive statistics were performed to obtain the mean, standard deviation, minimum, and maximum values of the measured body and liver mass and volume. The Kolmogorov–Smirnov test assessed the data’s normality, and the Levene test verified homoscedasticity. The Student’s *t*-test for independent samples was used to compare means between groups. The same normality and homoscedasticity tests were applied to the quantitative collagen fiber and stereological data. The Mann–Whitney U test was used to compare medians for type I collagen fibers, while the Student’s *t*-test was used for type III collagen fibers. For stereological data, means between groups were compared using a one-way analysis of the variance of general linear models. A value of *p* < 0.05 was considered statistically significant. All analyses were performed using the IBM SPSS Statistics software, version 21.

## 5. Conclusions

In conclusion, the liver of senescent rats exhibits a loss of normal tissue architecture with alterations in the arrangement of hepatocytes and the formation of cellular clusters. Inflammatory lesions and fibrosis predominantly occupying the portal space are observed in aging rats. Interlobular bile ducts in senescent rats show morphological changes compatible with the initial stage of primary biliary cholangitis. In senescence, there is an increase in the size of hepatocyte nuclei and a reduction in their volume density and number. The morphological and histopathological alterations found in the liver of senescent rats are consistent with processes involved in age-related chronic liver diseases in humans. These findings enhance our understanding of the mechanisms involved in hepatic aging and their significance in investigating human chronic liver diseases. They may also provide guidance for the creation of more efficient therapeutic approaches for the aging population.

## Figures and Tables

**Table 1:** Quantifying collagen content in liver tissue from adult (A) versus senescent (S) male Sprague Dawley rats using Image Pro Premier.

Collagen	Median ± SD		*p*
	Group A	Group S	
Type I (µm^2^)	18.18 ± 3.23	24.63 ± 9.99	0.001 *
Type III (µm^2^)	15.22 ± 3.02	15.75 ± 3.74	0.541

* Significant differences (*p* < 0.05).

**Table 2:** Comparison of body mass, liver mass, and volume in adult (A) versus senescent (S) male Sprague Dawley rats.

Variables	Group A		Group S		*p*
	Mean ± SD	Min–Max	Mean ± SD	Min–Max	
Body mass (g)	462.17 ± 46.21	401.00–519.00	366.73 ± 45.84	302.00–415.19	0.005 *
Liver mass (g)	20.85 ± 3.77	14.87–26.21	15.51 ± 1.40	13.98–18.03	0.009 *
Liver volume (cm^3^)	19.00 ± 3.46	14.00–24.00	14.33 ± 1.63	12.00–17.00	0.014 *

* Significant differences (*p* < 0.05).

**Table 3:** Stereological analysis of the liver of adult (A) versus senescent (S) male Sprague Dawley rats.

Parameters	Mean ± SD		*p*
	Group A	Group S	
Vv_hepM_ (%)	10.85 ± 2.52	8.65 ± 2.18	0.001 *
Vv_hepB_ (%)	1.01 ± 1.40	0.98 ± 1.62	0.086
Sv_hepM_ (mm^−1^)	27.78 ± 7.37	44.05 ± 11.39	0.001 *
Nv_hepM_ (mm^−3^)	177,579.37 ± 42,953.28	103,174.60 ± 45,478.43	0.001 *

* Significant differences (*p* < 0.05).

**Table 4:** Simple scoring system for chronic hepatitis modified from Scheuer.

Grade	
**Portal inflammation and interphase hepatitis**	
0	Absent or minimal.
1	Portal inflammation only.
2	Mild or localized interface hepatitis.
3	Moderate or more extensive interface hepatitis.
4	Severe and widespread interface hepatitis.
**Lobular activity**	
0	None.
1	Inflammatory cells but no hepatocellular damage.
2	Focal necrosis or apoptosis.
3	Severe hepatocellular damage.
4	Damage includes bridging confluent necrosis.
**Stage**	
0	No fibrosis.
1	Fibrosis confined to portal tracts.
2	Periportal or portal–portal septa but intact vascular relationships. Intact vascular relationships.
3	Fibrosis with distorted structure but no obvious cirrhosis. (septal fibrosis, bridging fibrosis).
4	Probable or definite cirrhosis.

**Table 5:** Stages of Scheuer’s Primary Biliary Cholangitis.

Stage	Rating
1	The florid duct lesion, portal hepatitis. *
2	Ductular reaction and periportal hepatitis.
3	Scarring, bridging necrosis, septal fibrosis.
4	Cirrhosis.

* Three components present: inflammation, damage of the ductal epithelium, and disruption of the bile duct basement membrane.

## Data Availability

Los datos se encuentran en este artículo. Los datos y materiales sin procesar son análisis realizados con el software IBM SPSS Statistics, versión 21, disponible a pedido de los autores.
